# Increasing Live Birth Rate by Preimplantation Genetic Screening of Pooled Polar Bodies Using Array Comparative Genomic Hybridization

**DOI:** 10.1371/journal.pone.0128317

**Published:** 2015-05-29

**Authors:** Michael Feichtinger, Tina Stopp, Christian Göbl, Elisabeth Feichtinger, Enrico Vaccari, Ulrike Mädel, Franco Laccone, Monika Stroh-Weigert, Markus Hengstschläger, Wilfried Feichtinger, Jürgen Neesen

**Affiliations:** 1 Wunschbaby Institut Feichtinger, Vienna, Austria; 2 Department of Obstetrics and Gynecology, Medical University of Vienna, Vienna, Austria; 3 Institute of Medical Genetics, Medical University of Vienna, Vienna, Austria; Inner Mongolia University, CHINA

## Abstract

Meiotic errors during oocyte maturation are considered the major contributors to embryonic aneuploidy and failures in human IVF treatment. Various technologies have been developed to screen polar bodies, blastomeres and trophectoderm cells for chromosomal aberrations. Array-CGH analysis using bacterial artificial chromosome (BAC) arrays is widely applied for preimplantation genetic diagnosis (PGD) using single cells. Recently, an increase in the pregnancy rate has been demonstrated using array-CGH to evaluate trophectoderm cells. However, in some countries, the analysis of embryonic cells is restricted by law. Therefore, we used BAC array-CGH to assess the impact of polar body analysis on the live birth rate. A disadvantage of polar body aneuploidy screening is the necessity of the analysis of both the first and second polar bodies, resulting in increases in costs for the patient and complex data interpretation. Aneuploidy screening results may sometimes be ambiguous if the first and second polar bodies show reciprocal chromosomal aberrations. To overcome this disadvantage, we tested a strategy involving the pooling of DNA from both polar bodies before DNA amplification. We retrospectively studied 351 patients, of whom 111 underwent polar body array-CGH before embryo transfer. In the group receiving pooled polar body array-CGH (aCGH) analysis, 110 embryos were transferred, and 29 babies were born, corresponding to live birth rates of 26.4% per embryo and 35.7% per patient. In contrast, in the control group, the IVF treatment was performed without preimplantation genetic screening (PGS). For this group, 403 embryos were transferred, and 60 babies were born, resulting in live birth rates of 14.9% per embryo and 22.7% per patient. In conclusion, our data show that in the aCGH group, the use of aneuploidy screening resulted in a significantly higher live birth rate compared with the control group, supporting the benefit of PGS for IVF couples in addition to the suitability and effectiveness of our polar body pooling strategy.

## Introduction

The success of an infertility treatment is strongly associated with the age of the female partner, mainly due to the rapid increase in aneuploidies that occurs in the oocytes of women aged 35 years and older. Additionally, aneuploidy rates in the oocytes of infertile female patients seem to be even higher than those in the oocytes of women of the same age without fertility problems [1,2]. Therefore, it is reasonable to assume that the identification of such oocytes or embryos without chromosomal aberrations in women over 35 years of age may improve pregnancy rates and consequently, live birth rates. Unfortunately, no consistent relationship appears to exist between the embryo karyotype and its morphology [3].

The technique of preimplantation genetic diagnosis (PGD) has been used for several years with the goal of either improving pregnancy rates by selecting euploid embryos or detecting specific genetically inherited diseases [4,5]. Since the introduction of PGD, a variety of different techniques have been developed for a wide range of indications [6–8]. The first attempts to analyze embryonic karyotypes used fluorescence *in situ* hybridization (FISH) to screen polar bodies, blastomeres or trophectoderm cells. However, several studies using FISH for aneuploidy screening have failed to show a clear benefit for women of advanced maternal age (AMA) or with recurrent implantation failure [9–11]. The limited number of chromosomes that can be examined by FISH is the most likely explanation for this lack of benefit. The analysis of complete embryo karyotypes has been achieved following the introduction of new techniques, such as comparative genomic hybridization (CGH), array-CGH, real-time PCR, and more recently, next-generation sequencing (NGS) [12,13]. Using these techniques, several studies have demonstrated improved pregnancy rates by screening all 24 chromosomes [14–16]. A large majority of these studies have applied array-CGH technology in combination with bacterial artificial chromosome (BAC) arrays.

As an alternative approach to the aneuploidy screening of blastomere or trophectoderm cells, the analysis of polar bodies by array-CGH has been discussed [17]. One disadvantage of polar body preimplantation genetic screening (PGS) is the high costs that arise because of the requirement for separate analyses of the first and second polar bodies to obtain a precise prediction of the putative chromosomal aberration in the oocyte. To reduce the costs of polar body analysis, we performed BAC array-CGH using DNA that was extracted and amplified from pooled polar bodies. Our results indicate that meiotic separation errors can be effectively detected in pooled polar bodies. Moreover, the live birth rate per transferred embryo strongly increased in couples after the BAC array-CGH-based PGS of pooled polar bodies in comparison with a control IVF group without PGS.

## Methods

In the present study, 351 women between 35 and 45 years of age were included. The patients were treated using standard IVF/ICSI protocols. In the study group (aCGH group), 111 patients with a mean age of 39.5 years underwent BAC array-CGH-based aneuploidy screening (PGS) before embryo transfer using DNA obtained from pooled polar bodies. The indication for polar body screening was either repeated implantation failure or advanced maternal age.

The control group without PGS before embryo transfer consisted of 240 consecutive patients who were also between 35 and 45 years of age (mean age of 38.4 years). Intracytoplasmatic sperm injection (ICSI) was performed for 231 patients and oocytes of nine patients (3.8%) were fertilized by in vitro fertilization (IVF). Each patient was included only once in the study, and for women who underwent more than one IVF/ICSI attempt during the recruitment time, only the last treatment was taken into account for the present study. In both groups, 1 to 3 embryos were transferred between days 2 and 5. In the aCGH group, only embryos for which the screening indicated no chromosomal aberration were used for transfer, while in the control group, the embryos for transfer were chosen on the basis of morphological criteria [18,19]. The primary endpoints were the live birth rates for both groups.

All patients signed an informed consent form. The present case-control study was performed according to the STROBE guidelines [20] and was approved by the institutional review board of the Medical University of Vienna.

### Polar body biopsy

In the aCGH patient group, two to three hours after oocyte retrieval, cumulus cells were dissolved using hyaluronidase medium. Intracytoplasmatic sperm injection (ICSI) was performed for all patients in the aCGH group to avoid contamination. Biopsies of both polar bodies were conducted at 16 to 18 hours after ICSI. Assisted hatching of the embryo was performed using an OCTAX laser system (Octax Microsience GmbH, Bruckberg, Germany).

### DNA amplification of polar bodies

First and second polar bodies were transferred together into a 0.2-ml microtube containing medium with 2.5 μl of phosphate-buffered saline (PBS). Extraction and amplification of the DNA from the polar bodies was performed according to the BlueGnome SurePlex CGH Amplification System protocol (SurePlex; BlueGnome, Cambridge, UK). After DNA amplification, 5-μl aliquots of the products were separated by gel electrophoresis as a quality control measure, and 1-μl aliquots were used for DNA quantification with a Qubit 2.0 Fluorometer (Life Technologies, Vienna, Austria). Typically, WGA amplification resulted in a DNA concentration of 20–40 ng/μl.

### Array-CGH analysis

Eight microliters of the amplified DNA from the pooled polar bodies as well as male and female control DNA were labeled according to the 24sure V3 protocol (BlueGnome, Cambridge, UK). Either Cy3 dCTP or Cy5 dCTP nucleotides were incorporated into the DNA using random primers and Klenow enzyme. A labeling reaction was performed for two hours in a thermal cycler at 37°C. Thereafter, the samples were dried in a centrifugal evaporator at 75°C for 40 minutes. After the addition of 25 μl of COT human DNA (1 μg/μl) to the probes, the probes were again incubated in a centrifugal evaporator at 75°C until the volume was reduced to approximately 3 μl. The DNA was then resolved and denatured at 75°C for 10 minutes in 21 μl of hybridization buffer containing 15% dextran sulfate. Eighteen microliters of the DNA solution was used for hybridization to the BAC array for 4 to 16 hours at 47°C. The washing steps were performed according to the 24sure protocol. Finally, the slides were dried by centrifugation at 200 x *g* for 2 minutes and scanned using a DNA Microarray Scanner (Agilent Technologies, Santa Clara, United States) at 10 μm resolution. Signals were called with BlueGnome software (BlueFuse Multi analysis software version 3.0), with an adjustment for first polar body analysis [16]. Aneuploidy screening using BlueFuse Multi analysis software is based on the median log_2_ ratio for each chromosome. For analysis of a diploid cell, the predicted log_2_ ratio for chromosome gain is +0.58 (log_2_ 3/2), and for chromosome loss, it is -1.0 (log_2_ ½). For pooled polar bodies, the predicted log_2_ ratio for chromatid gain is reduced to +0.48 (log_2_ 4/3), and for chromatid loss, it is -0.59 (log_2_ 2/3). BlueFuse Multi analysis software is able to detect a chromosome gain with 100% confidence when the median ratio is ≥ +0.35 and a chromosome loss with 100% confidence when the median ratio is ≤ -0.6 [21,22].

### Multiplex STR analysis

Multiplex STR analysis using a PowerPlex 16 System (Promega, Mannheim, Germany) was performed to verify the successful transfer of the first and second polar bodies to the tube, to assess the possible contamination of the probes and to determine the ratio of DNA amplification between the two polar bodies in a random subset of the aCGH group. Forty samples were tested using 1 μl of amplified DNA from pooled polar bodies. Amplification of the polymorphic DNA fragments was conducted according to the manufacturer's protocol. One microliter of the product was separated with an ABI PRISM 3170 Avant Genetic Analyzer, and fragment sizes were determined using Gene Mapper software (Life Technologies, Vienna, Austria).

### Statistical analysis

Continuous variables were presented as the mean ± standard deviations (SD), and categorical variables were represented by counts and percentages, unless indicated otherwise. Group-based comparisons including all samples were compared using Student’s *t*-test (or the Wilcoxon rank sum test if the normality assumption was violated) or Fisher's exact test. Uni- and multivariable logistic regression models were used to assess associations between the outcome parameter (i.e., live birth) and explanatory variables, such as polar body analysis (the parameter of main interest), age (metric scaled, years), BMI (metric scaled, kg/m^2^), the number of treatment attempts >1 (binary), the number of oocytes (square root-transformed (sqrt)), the number of transferred embryos >1 (binary), and the day of transfer (categorical). A backward stepwise selection algorithm was used for variable reduction in the multivariable logistic regression model, whereby the selection algorithm was applied according to Akaike’s information criterion. Effects were expressed as odds ratios (ORs) and 95% confidence intervals (95% CIs). Statistical analysis was performed with R (V3.0.2) and associated packages [23] and SPSS (version 21). A two-sided p-value of ≤0.05 was considered to be statistically significant. P-values were interpreted descriptively, and multiplicity was not adjusted in this observational study.

## Results

### STR analyses

In a first step to establish aneuploidy screening using amplified DNA from pooled polar bodies, we performed multiplex analyses on 40 DNA samples, testing 16 highly polymorphic STR markers. DNA samples of pooled first and second polar bodies should contain three chromatids for each chromosome, and for a heterozygous allele, a 2:1 ratio should be observed. Uneven amplification of the DNA from the first and second polar bodies by a WGA kit would interfere with this 2:1 distribution. Moreover, contamination of the polar body sample by extrinsic DNA should result in the detection of more than two alleles for at least one of the tested STR markers. In all 40 analyzed samples, we observed two or more STR markers displaying a 2:1 distribution, supporting the successful transfer of both polar bodies to the tube as well as the proportionate DNA amplification of all three chromatids. Additional STR signals were not observed, indicating that the contamination of the samples by exogenous DNA or cells was unlikely.

### Array-CGH analyses

**Fig 1:**
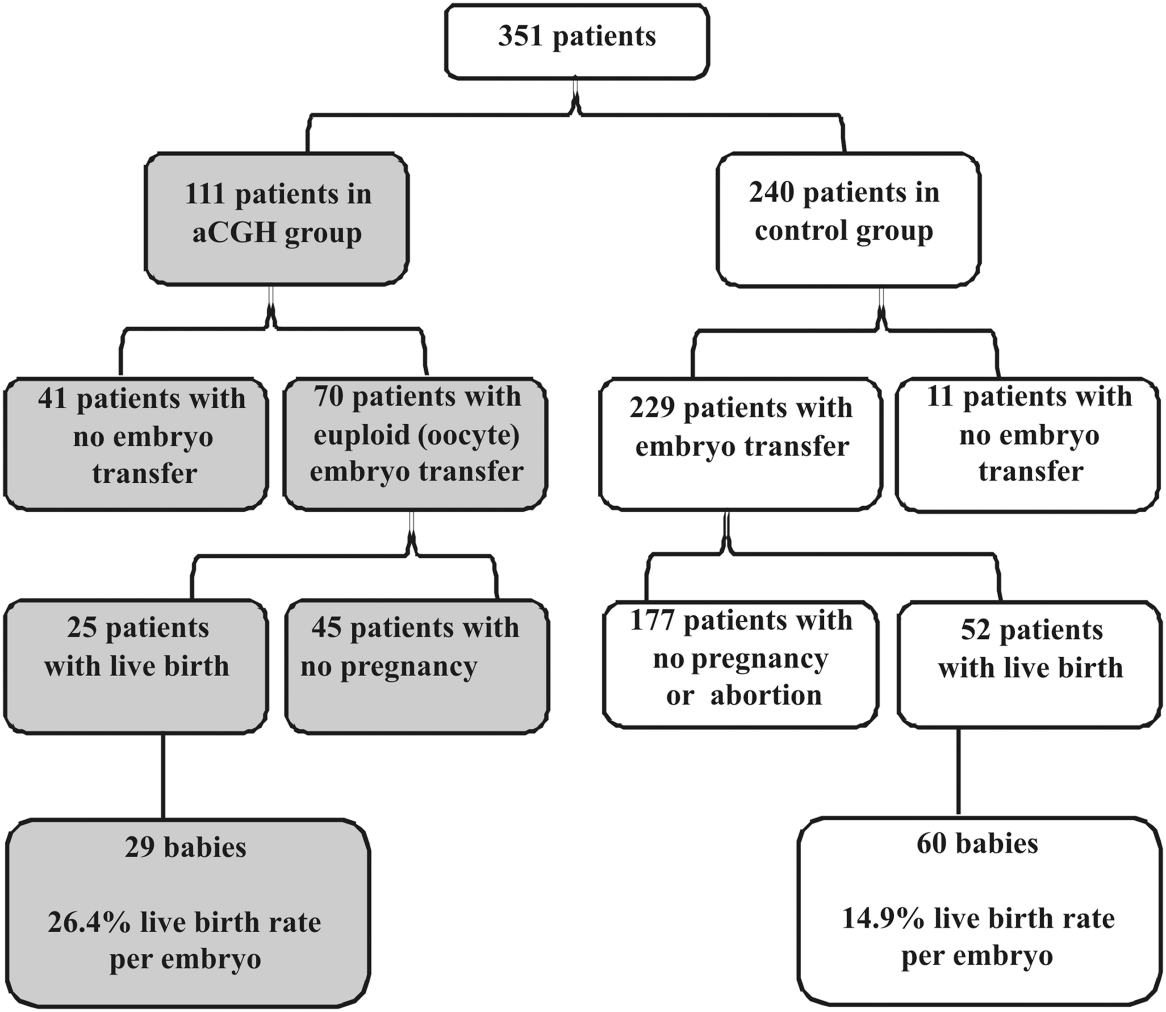
Flowchart of patients’ treatments. The white fields show data for the control group, whereas the gray shadowed fields depict the data for the array-CGH (aCGH) group. In the aCGH group, 41 patients had no embryo transfer because no euploid oocyte could be identified. In the control group, 11 patients had no embryo transfer because no oocyte could be retrieved or fertilized or because of poor embryo quality.

Of the 351 patients included in the study, 111 (32%) received BAC array-CGH-based aneuploidy screening of pooled polar bodies, while 240 (68%) did not (Fig 1). Descriptive analysis of all samples is provided in Table 1. The mean age of the women in the aCGH group was 39.5 years, while in the control group, the mean age was 38.4 years. The women in the aCGH group had a significantly greater number of previous IVF/ICSI attempts (Table 1). There were no significant differences in the IVF/ICSI protocols between the two groups (p = 0.205); however, more embryos were transferred in the control group, with an average of 1.76 embryos, compared to a mean transfer of 1.57 embryos in the group subjected to aCGH analysis.

**Table 1:** Characteristics of the total study population.

	non-CGH	CGH	p-value
	n = 240	n = 111	
Age (years)	38.4(yea	39.5(yea	<0.001
Antagonist protocol	152 (63.3%)	73 (65.8%)	0.394
Agonist protocol	87 (36.3%)	38 (34.2%)	0.394
BMI (kg/m^2^)	20.3(kg/	20.5(kg/	0.354
Attempts	2.0 (1.0–3.0)	2.0 (1.0–4.0)	0.002
Attempts >1	142 (59.2%)	78 (70.2%)	0.046
Number of oocytes	6.0 (4.0–10.0)	8.0 (5.0–11.0)	<0.001
Transferred embryos	2.0 (1.0–2.0)	1.0 (0.0–2.0)	<0.001
Transferred embryos >1	154 (65.0%)	48 (43.2%)	<0.001
Day of embryo transfer	3.0 (2.0–3.0)	3.0 (3.0–5.0)	0.070

The data are expressed as the mean ± standard deviation as well as the median (IQR) and counts (%).

* p-value based on the Wilcoxon rank sum test.

**Fig 2:**
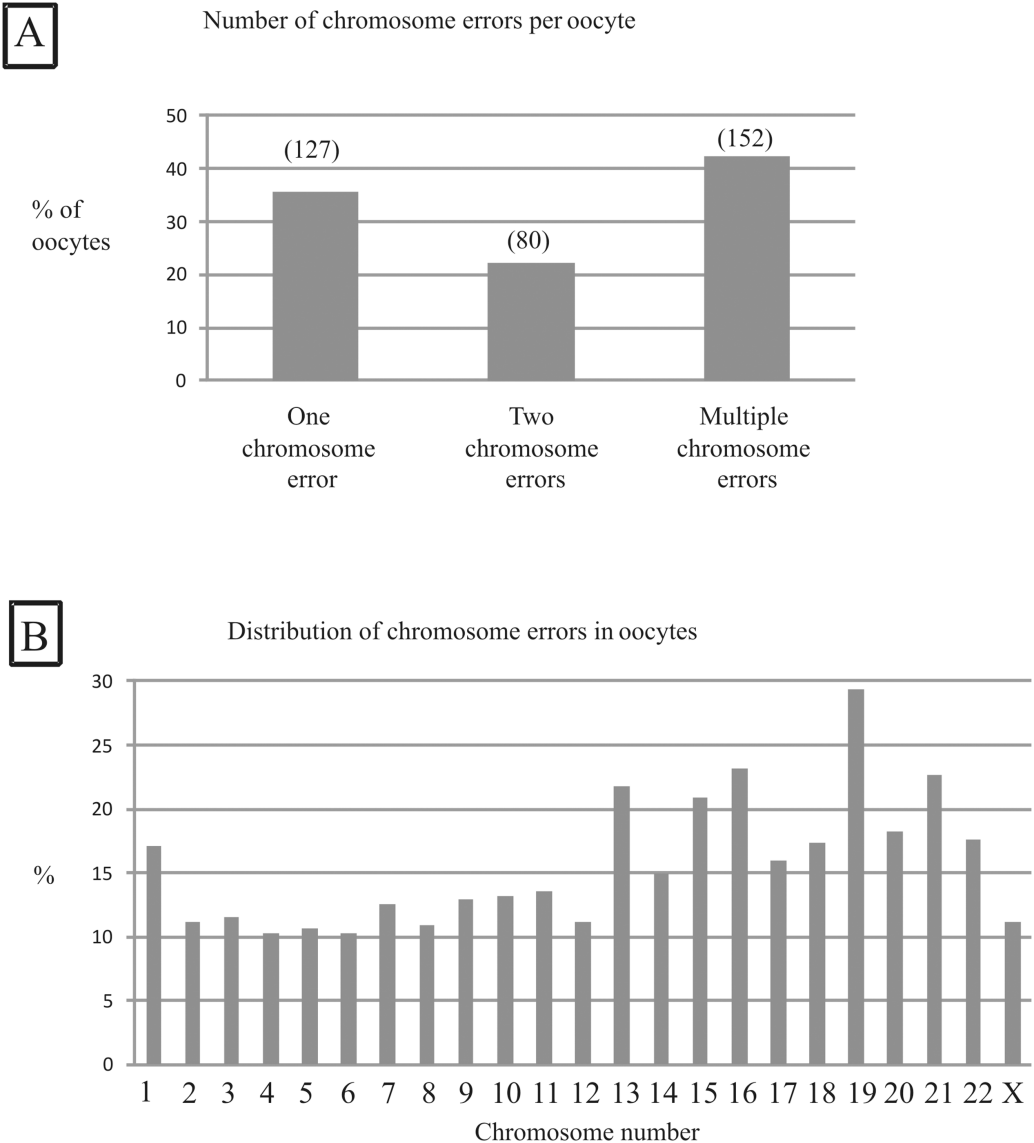
(A) Number of chromosomal aberrations in aneuploid oocytes. In total, the polar bodies of 530 oocytes were tested by aCGH, and 359 were found to have a chromosomal aberration. Approximately 65% of aneuploid oocytes had two or more aneuploidies. (B) Distribution of chromosome errors in aneuploid oocytes. All chromosomes were found to be involved in aneuploidies. Aneuploidy of chromosome 4 was observed in only 10% of oocytes, while chromosome 19 aberrations were most frequently detected in up to 30% of oocytes.

In the aCGH group, a total of 930 oocytes were collected. Polar bodies were isolated from 530 oocytes and analyzed by aCGH, while the other oocytes did not develop or polar bodies could not be isolated from them. The results of the polar body screening revealed that 147 (28.6%) oocytes were euploid, whereas 359 (67.7%) were not. No results were obtained for 24 oocytes (4.5%). In 35.6% of aneuploid oocytes, a gain or loss of only one chromosome was observed (Fig 2A). Gains or losses of two chromosomes were observed in 22.2% of aneuploid oocytes, and 42.2% of aneuploid oocytes showed three or more chromosomal aberrations (Fig 2A). All 23 chromosomes were involved in aneuploidies. The gain or loss of chromosome 19 was the most commonly detected aneuploidy (28.7%), whereas aneuploidy for chromosome 4, 5 or 6 was observed scarcest affecting approximately 10% of aneuploid oocytes (Fig 2B). Forty-one patients (37%) had no oocytes without a putative chromosome aberration, and these women decided not to undergo embryo transfer (Fig 1). In the control group without aCGH, embryo transfer was not performed in eleven patients (4.6%). Overall, 52 women did not undergo transfer. Therefore, the remaining 299 patients were considered for further analysis.

In the control group, a total of 403 embryos were transferred, and 60 babies were born, corresponding to live birth rates of 14.9% per embryo and 22.7% per patient. In the aCGH group, 110 embryos were transferred, and 29 babies were born, resulting in a significantly higher live birth rate of 26.4% per embryo (p = 0.015) and a live birth rate of 35.7% per patient (p = 0.031). In addition, in the aCGH group, no women with clinical pregnancy experienced a miscarriage, while in the control group, nine patients (3.93%) lost their fetus.

Interestingly, in the control group, only two babies were born from women who were 40 and 41 years old, while in the aCGH group, 9 babies were born from women who were 40 to 43 years old, suggesting a benefit of the aCGH analysis, especially for women of an advanced age (Table 2).

**Table 2:** Results of the IVF-treatment in combination with or without aCGH.

Age	No. of patients	No. of transferredembryos	No. of live births	No. of twin births	No. of tested oocytes	No. of euploid oocytes	% of euploid oocytes
35	34	51	12	1			
	5	9	4	1	32	18	56.3
36	34	56	10	2			
	6	3	-	-	30	3	10.0
37	27	52	12	2			
	11	14	4	-	58	21	36.2
38	40	67	13	2			
	13	16	7	2	63	28	44.4
39	36	63	11	1			
	23	23	5	1	111	41	36.9
40	27	47	1				
	21	18	2	-	85	27	31.8
41	12	21	1				
	10	13	4	-	47	14	29.8
42	7	9	-				
	9	5	1	-	32	8	25.0
43	12	19	-				
	8	6	2	-	41	9	22.0
44	7	12	-				
	3	1	-	-	10	1	10.0
45	4	6	-				
	2	2	-	-	11	2	18.2

The first column depicts the patients’ ages. In each line upper rows show the data for the control group, and the lower rows show the data for the aCGH group.

A univariable analysis revealed that the age of the patient, number of transferred oocytes, transfer of more than one embryo, day of transfer and aCGH were associated with the live birth rate (Table 3). In addition, multivariable logistic regression models were used to adjust for several possible confounders, revealing that the live birth rate was significantly higher in the polar body analysis group after adjustments for the remaining variables (Table 3). Moreover, aCGH (OR 2.57, 95% CI 1.37–4.82, p = 0.003), age (OR 0.80, 95% CI 0.70–0.91, p<0.001), and the transfer of >1 embryo (OR 2.15, 95%CI 1.19–4.01, p = 0.013) were selected as independent predictors of the live birth rate by backward stepwise elimination.

**Table 3:** Uni- and multivariable analyses of 299 subjects with transfers resulting in live births.

		Univariable			Multivariable*	
	OR	95% CI	p-value	AOR	95% CI	p-value
CGH	1.89	1.05–3.36	0.031	2.83	1.36–5.96	0.006
Age (years)	0.83	0.74–0.93	0.002	0.79	0.69–0.91	0.001
BMI (kg/m^2^)	1.00	0.94–1.07	0.885	1.01	0.98–1.05	0.473
Attempts >1	0.87	0.51–1.49	0.596	0.85	0.47–1.54	0.583
Sqrt (oocytes)	1.44	1.04–2.01	0.030	1.01	0.65–1.53	0.980
Embryos >1	1.97	1.11–3.60	0.023	2.14	1.15–4.11	0.018
Day of ET = 1 or 2 (reference)	-	-	-	-	-	-
Day of ET = 3	1.01	0.52–1.97	0.982	0.85	0.41–1.79	0.666
Day of ET = 4	1.09	0.28–3.49	0.897	0.66	0.15–2.46	0.559
Day of ET = 5	2.13	1.06–4.36	0.035	1.32	0.55–3.20	0.537

*Adjusted odds ratios (AORs) for a fully adjusted logistic regression model.

## Discussion

The results of this study strongly suggest that array-CGH analysis using amplified DNA from pooled polar bodies improves the live birth rate compared with that observed in the absence of aneuploidy screening. A number of studies have demonstrated that chromosome aberrations are strongly increased in women older than 35 years and that aneuploidy may be the main reason for infertility in couples with an advanced maternal age. Aneuploid oocytes can be identified with high sensitivity by the comprehensive chromosome screening of amplified DNA from polar bodies. In former studies, polar bodies have been evaluated to predict the majority of aneuploidies in resulting embryos [24,25]. However, in these studies, first and second polar bodies were extracted successively from oocytes and amplified separately. After hybridization, the polar bodies were analyzed using software with specific settings for the first and second polar bodies. Because the BlueFuse Multi software that we used has no setting for pooled polar bodies, we plotted our samples with an adjustment for first polar body analysis. Previous studies have shown that BlueFuse Multi software is able to detect mosaicism for aneuploidy at levels as low as 25–37% with high confidence [21,22]. Our approach to pool the first and second polar bodies corresponds to an aneuploidy mosaicism of 50–60%. Therefore, if the quality of the hybridization signals is sufficient, the BlueFuse Multi software will detect chromatid gains or losses in pooled polar bodies with high efficiency. However, it should be noted that a certain amount of false positives have been reported in previous studies involving sequential and complementary assessments of polar bodies [25]. These discordant results may be due to trisomic rescue, which occurs in the embryo at a very early stage of cleavage and can result in either a normal embryo or an embryo with uniparental disomy or isodisomy [24–27]. Depending on the involved chromosome, disomy is expected to have clinical consequences in the child. In contrast with polar body analysis, isodisomic embryos can be identified by analysis of trophectoderm cells using SNP arrays.

Our results show that approximately two-thirds of aneuploid oocytes have copy number variations of two or more chromosomes, making a rescue resulting in an euploid embryo quite unlikely. In fact, another study has shown that only 1% of embryos of AMA patients found to be aneuploid by polar body analysis result in an embryo without a chromosomal aberration [24,26,28,29]. Reciprocal aneuploidy may be another reason for obtaining false positive or false negative results in sequential polar body analysis. Depending on the quality of the experiment, it may be difficult to distinguish between a gain/loss of a single chromatid versus the gain/loss of a chromosome with two chromatids and to assess a chromosomal imbalance. Therefore, in several studies putative euploid embryos were lost because in the presence of reciprocal chromosome aberrations in first and second polar bodies these embryos were predicted as aneuploid [30]. Our approach using pooled polar bodies limits the risk of the misinterpretation of putative reciprocal chromosomal aberrations in the first and second polar bodies. This assumption is supported by the results of our multiplex STR analyses, which suggested that DNA amplification of the first and second polar bodies was balanced, although we could not completely rule out the asymmetric amplification of partial DNA from the first or second polar body occurring in the same reaction.

Another potential cause of false results in aneuploidy screening using DNA from pooled polar bodies can be an undetected loss of one polar body during biopsy [31–33]. However, for the prediction of oocyte karyotypes, the analysis of both polar bodies is essential because chromosomal aberrations may occur during the first or second meiotic division [34]. Multiplex STR analysis can be used to verify the successful transfer of both polar bodies as well as to exclude contamination of the sample with external DNA.

A clear disadvantage of polar body analysis versus a later analysis of embryonal cells is the inability to detect aneuploidies of paternal origin. However, paternal aneuploidies reflect only 3–4% of numeric chromosomal aberrations [1,35,36]; furthermore, no increase in aneuploidy has been detected in men of advanced paternal age [37]. This finding applies to couples with a normospermic male partner; however, the risk of paternal-derived aneuploidy may be increased in men with spermatogenic defects [25]. Whether women of advanced maternal age having a partner with a spermatogenic defect may also benefit from PGS using DNA from pooled polar bodies must be evaluated.

In blastomere biopsies, mosaicism of the pre-embryo presents a diagnostic challenge in selecting euploid embryos. Up to 24% of blastomere biopsies have been shown to be false positives due to the biopsy of an aneuploid cell from a blastomere in which the remainder is euploid [38]. On the other hand, 22% of blastomere biopsies have been shown to be false negatives, indicating the aspiration of a euploid cell from a blastomere in which the remainder is aneuploid, leading to incorrect procedural decisions [38]. The issue of mosaicism questions the diagnostic accuracy of the analysis of one blastomere cell as a representation of the whole embryo. However, mosaicism does not impact the diagnosis of aneuploidy in polar bodies, and PGS has been shown to have over 90% diagnostic efficiency when aCGH is applied [39]. Additionally, the removal of more than one cell from a cleavage stage embryo to enhance diagnostic security significantly reduces the implantation rate [40]. Even the removal of one blastomere has been shown to diminish the implantation potential by 12.5% [41]. The effect of polar body biopsy on the implantation rate has not been studied to date. The aneuploidy rates of blastocysts and polar bodies appear to be very similar, while blastomeres have an altered rate of aneuploidy, possibly caused by mitotic errors [29,42,43]. These mitotic origins of aneuploidy cannot be assessed by polar body analysis.

Most women of advanced maternal age experience a decline in oocyte numbers and consequently, a reduced number of blastocysts, causing the preimplantation genetic screening of blastocysts to be difficult in this group [44,45]. PGS of DNA from pooled polar bodies may be an alternative for these women to avoid the manipulation of the blastocyst. Our results suggest that euploid embryos can be identified in woman of advanced maternal age, improving the chances of having a live birth.

Recently published studies have revealed that the most frequent aneuploidies affect chromosomes 16, 21, 22, 15 and 19, representing more than half of all aneuploidies [29]. Our results are very similar, indicating that chromosome 19 is the most frequent chromosome affected by aneuploidy, followed by chromosomes 16, 21, 13, 15 and 22. Conventional FISH (fluorescence *in situ* hybridization) analysis does not cover all chromosomes; therefore, a significant amount of fatal aneuploidies cannot be detected, and this technology is not recommended for PGS [29,34,46]. In contrast, classical comparative genomic hybridization provides information on all chromosomes contained in the oocyte; however, this technique is labor-intensive [47]. Therefore, conventional CGH has been replaced by array-CGH, which can be automated and allow for the detection of chromosomal aberrations with a very high level of accuracy [48]. By applying aCGH to evaluate embryos on days 3 and 5, the deleterious effects of maternal age on pregnancy and the implantation rate may be diminished [49].

In some countries, preimplantation diagnosis of embryos has legal implications that may be avoided by analyzing polar bodies. Even for patients who oppose conventional preimplantation diagnosis of the early embryo for religious reasons, polar body analysis may represent an alternative approach [50,51]. Because polar body biopsy can be performed on the day of fertilization, there is adequate time for analysis, and embryo freezing can be avoided [52]. However, performing conventional polar body aneuploidy screening during the early stage of embryonic development leads to increased costs for IVF couples because more samples need to be analyzed. Our results demonstrate that the first and second polar bodies can be pooled for PGS without a significant loss of sensitivity, notably reducing the costs of analysis. In our study, 36.9% of the patients who underwent polar body analysis had no embryo transfer because none of the retrieved oocytes were found to be euploid. This situation represents a negative factor influencing the outcome of assisted reproductive technology [53]. However, these patients do not need to undergo psychological pressure waiting for a very unlikely positive pregnancy test or to experience the stress of a very probable pregnancy loss.
