# Correction: Increasing Live Birth Rate by Preimplantation Genetic Screening of Pooled Polar Bodies Using Array Comparative Genomic Hybridization

**DOI:** 10.1371/journal.pone.0133334

**Published:** 2015-07-15

**Authors:** Michael Feichtinger, Tina Stopp, Christian Göbl, Elisabeth Feichtinger, Enrico Vaccari, Ulrike Mädel, Franco Laccone, Monika Stroh-Weigert, Markus Hengstschläger, Wilfried Feichtinger, Jürgen Neesen

There are errors in Table 1. Please see the correct Table 1 here.

**Table 1:** Characteristics of the total study population.

	non-CGH	CGH	p-value
	n = 240	n = 111	
Age (years)	38.4±2.5	39.5±2.3	<0.001
Antagonist protocol	152 (63.3%)	73 (65.8%)	0.394
Agonist protocol	87 (36.3%)	38 (34.2%)	0.394
BMI (kg/m^2^)	20.3±9.2	20.5±7.6	0.354
Attempts	2.0 (1.0–3.0)	2.0 (1.0–4.0)	0.002
Attempts >1	142 (59.2%)	78 (70.2%)	0.046
Number of oocytes	6.0 (4.0–10.0)	8.0 (5.0–11.0)	<0.001
Transferred embryos	2.0 (1.0–2.0)	1.0 (0.0–2.0)	<0.001
Transferred embryos >1	154 (65.0%)	48 (43.2%)	<0.001
Day of embryo transfer	3.0 (2.0–3.0)	3.0 (3.0–5.0)	0.070

The data are expressed as the mean ± standard deviation as well as the median (IQR) and counts (%).

* p-value based on the Wilcoxon rank sum test.
